# Efficacy of Hydroxy-L-proline (HYP) analogs in the treatment of primary hyperoxaluria in *Drosophila Melanogaster*

**DOI:** 10.1186/s12882-018-0980-8

**Published:** 2018-07-06

**Authors:** Huan Yang, Musa Male, Yang Li, Ning Wang, Chenming Zhao, Shan Jin, Juncheng Hu, Zhiqiang Chen, Zhangqun Ye, Hua Xu

**Affiliations:** 1 Department of Urology, Tongji Hospital, Tongji Medical College, Huazhong University of Science and Technology, 1095# Jie Fang Avenue, Wuhan, 430030 China; 2 Institute of Urology, Tongji Hospital, Tongji Medical College, Huazhong University of Science and Technology, Wuhan, China; 3 College of Life Sciences, Hubei University, Wuhan, China

**Keywords:** Hydroxy-L-Proline analogs, Primary hyperoxaluria, *Drosophila Melanogaster*

## Abstract

**Background:**

Substrate reduction therapy with analogs reduces the accumulation of substrates by inhibiting the metabolic pathways involved in their biosynthesis, providing new treatment options for patients with primary hyperoxalurias (PHs) that often progress to end-stage renal disease (ESRD). This research aims to evaluate the inhibition efficacy of Hydroxy-L-proline (HYP) analogs against calcium oxalate (CaOx) crystal formation in the *Drosophila Melanogaster* (*D. Melanogaster*) by comparing them with Pyridoxine (Vitamin B6).

**Methods:**

Three stocks of *Drosophila Melanogaster* (W^118^, CG3926 RNAi, and Act5C-GAL4/CyO) were utilized. Two stocks (CG3926 RNAi and Act5C-GAL4 /CyO) were crossed to generate the Act5C > dAGXT RNAi recombinant line (F_1_ generation) of *D. Melanogaster* which was used to compare the efficacy of Hydroxy-L-proline (HYP) analogs inhibiting CaOx crystal formation with Vitamin B_6_ as the traditional therapy for primary hyperoxaluria.

**Results:**

Nephrolithiasis model was successfully constructed by downregulating the function of the dAGXT gene in *D. Melanogaster* (*P*-Value = 0.0045)*.* Furthermore, the efficacy of Hydroxy-L-proline (HYP) analogs against CaOx crystal formation was demonstrated in vivo using *D. Melanogaster* model; the results showed that these L-Proline analogs were better in inhibiting stone formation at very low concentrations than Vitamin B_6_ (IC_50_ = 0.6 and 1.8% for standard and dietary salt growth medium respectively) compared to N-acetyl-L-Hydroxyproline (IC_50_ = 0.1% for both standard and dietary salt growth medium) and Baclofen (IC_50_ = 0.06 and 0.1% for standard and dietary salt growth medium respectively). Analysis of variance (ANOVA) also showed that Hydroxy-L-proline (HYP) analogs were better alternatives for CaOx inhibition at very low concentration especially when both genetics and environmental factors are intertwined (*p* < 0.0008) for the dietary salt growth medium and (*P* < 0.063) for standard growth medium.

**Conclusion:**

Addition of Hydroxy-L-Proline analogs to growth medium resulted in the reduction of CaOx crystals formation. These analogs show promise as potential inhibitors for oxalate reduction in Primary Hyperoxaluria.

## Background

Nephrolithiasis is a major public health predicament with diverse and convoluted etiology. In humans, calcium oxalate (CaOx) is the primary component of nephrolithiasis which accounts for about 80% of all stones [1–5]. Thus, most of the investigations of nephrolithiasis have concentrated on CaOx stones. Many factors can induce nephrolithiasis, and these encompass acidic urinary pH, dehydration, hypercalciuria, particular medications, hyperoxaluria and hereditary disorders. Among these factors, hyperoxaluria is the gravest. Hyperoxaluria is either acquired or inherited [6, 7].

The primary hyperoxalurias (PHs) are autosomal recessive conditions with inborn metabolic defects that result in increased endogenous oxalate production by the liver leading to excessive urinary oxalate excretion [6–11]. Up to the present time, three distinct hereditary enzymatic deficiencies have been linked to PH, to be precise, PH type 1 (PH1) which results from mutations in peroxisomal enzyme alanine: glyoxylate aminotransferase (AGT; the AGXT gene product) [2, 12, 13], type 2 (PH2) which results from mutations in glyoxylate reductase (GR; the GRHPR gene product), and type 3 (PH3) which results from inactivating mutations in 4-hydroxy-2-oxoglutarate aldolase (HOGA; the HOGA1 gene product) [7].

Current treatments for PH are centered around renal function conservation, and these include increased fluid intake and chemicals to inhibit calcium oxalate Crystallization in the urine [9, 11, 13]. Alkali citrate is the most commonly used therapeutic modality which can alkalinize the urine and lower the urinary calcium oxalate saturation by forming complexes with calcium henceforth decreasing stone growth [14]. Oxalate-degradation bacteria (*Oxalobacter formigenes)* have been extensively studied on hyperoxalurias though with limited effects in PH patients. They can metabolize oxalate and thus may have a role in promoting intestinal oxalate excretion although a recent clinical trial reported disappointing results [15, 16]. Pyridoxine (vitamin B_6_) is conventionally used in the treatment of PH type 1 patients (especially Gly170Arg, Phe152Ile, and Ile244Thr genotypes) [5, 17]. However, high doses are typically required to reduce the production of oxalate through heightening the conversion of glyoxylate to glycine hence decreasing the amount of substrate accessible for metabolism to oxalate. Organ transplantation seems to have an excellent effect on the PH treatment, most especially the combined liver-kidney transplantation [5], but the enormous cost coupled with low survival rate after transplantation makes it limited. Combined liver/kidney transplant is the only effective treatment for reducing oxalate production in PH type 1 patients who do not respond to high-dose vitamin B_6_ therapy [18–20]. The ubiquitous tissue distribution of GRHPR in PH type 2 patients favors kidney transplantation although it is still limited [14]. Nonetheless, failure of isolated kidney transplantation in PH type 2 patients has been reported [21]. On the contrary, PH type 3 patients typically do not progress to end-stage renal disease (ESRD) [17, 22].

With these limitations, researchers and doctors are working tirelessly to find new treatments. Enzymes that can significantly degrade oxalate in vitro have been discovered, but instability makes them unsuitable for in vivo application. Moreover, recent studies on some compounds discovered various small molecules that could inhibit the formation of oxalate [13]. These inhibitors are analogs of proline which can inhibit the activity of proline dehydrogenase thereby decreasing the conversion of glyoxylate to oxalate. Although these inhibitors were reported to be very effective in vitro, no in vivo experiments were reported. Moreover, the reported inhibitors have certain toxicity to animals and humans henceforth more attention should be paid. The recent discovery of some side chain groups conversion was found lessen their toxicity [23, 24]. Nevertheless, there is no in vivo report about how effectively they can inhibit the formation of oxalate.

*Drosophila Melanogaster* has been successfully utilized in the studies of a multiplicity of human diseases traversing diverse organ systems. In their report, Chien and colleagues stated that in the cross-genomic analysis, more than 70% of human disease loci had been discovered to have their homolog in the *D. melanogaster* genome [25]. Furthermore, Miller and colleagues described the renal system of the *Drosophila melanogaster* to comprises the nephrocytes and Malpighian tubules which are two discrete organs anatomically and functionally [3, 4, 26]. The renal system is among the extremely conserved organ systems in the Drosophila, with dozens of the Drosophila genes that analogous to genetic disorders of the human kidney. The Malpighian tubules are analogous to the rest of the human nephron and collecting duct [27]. Nephrocytes are specialized groups of cells conglomerated proximal to the heart and the esophagus which filter the fly’s hemolymph (circulatory fluid) in addition to removing waste products in a manner comparable to the endocytic processes of podocytes in the human glomerulus. This versatile invertebrate is now emanating as a compelling translational model of human nephrolithiasis with a diversity of functional and pragmatic advantages.

Therefore, in this study, we selected N-acetyl-L-Hydroxyproline, Baclofen and Vitamin B_6_ to study their inhibitory effects on the formation of stones in vivo. W^1118^ (Wild-type), CG3926 RNAi (non-driven RNAi line) and Actin-GAL4/CyO (Housekeeping gene) adult fruit flies of *D. Melanogaster* were used as the model.

## Methods

### Insects and treatments

#### Insects

Adult fruit flies of *D. Melanogaster* of W^1118^ (#3605, Bloomington, USA), Actin-GAL4/CyO (#4414, Bloomington, USA) from Bloomington Drosophila Stock Center [https://bdsc.indiana.edu/] and CG3926 RNAi (#TH02225.N, Beijing) from Tsinghua Fly Center [http://fly.redbux.cn/rnai.php?lang=en] were used in these experiments. These flies were either fed with standard growth medium in this study to evaluate the relationship of dAGXT gene and CaOx crystal formation or with dietary salt growth medium containing 0.05% Sodium oxalate [http://en.reagent.com.cn/enshowproduct.jsp?id=10020118] which was used to investigate the exacerbation effects of extrinsic factors on the genetics as well as the efficacy of the inhibitors. In summary, flies were bred in plastic vials containing fly growth medium (standard or dietary salt) maintained under standard conditions at 25 °C and (40–60) % humidity with a twelve-hourly light-dark cycle [27, 28]. Every after 3 days, these flies were transferred to plastic vials containing new similar growth medium, and the cycle was repeated throughout the entire experiment.

The standard growth medium consisted of 15.0 g agar, 90.0 g brewer’s yeast, 189.6 g glucose, 94.86 g sugar, and 233.1 g cornmeal, with the addition of water to a final volume of 3.5 L. The solution was heated to boiling until a homogenous mixture was attained, and after cooling to below 60 °C, 3 mL ethanol, 1.7 g benzoic acid and 9 ml n-propionic acid were added. Then, 10 mL of medium decanted into plastic vials and left at room temperature to cool down before its storage.

#### Dietary salt growth medium

Sodium oxalate (NaOx) was dissolved in 100 ml of standard growth media (0.05% concentration was set [3]) just after its preparation and mixed, and the diet was left to set. The Diet (both standard and dietary salt growth medium) was freshly prepared prior to each transfer to avoid any changes in the concentration of the salts due to evaporation.

#### Inhibition studies

**Fig. 1:**
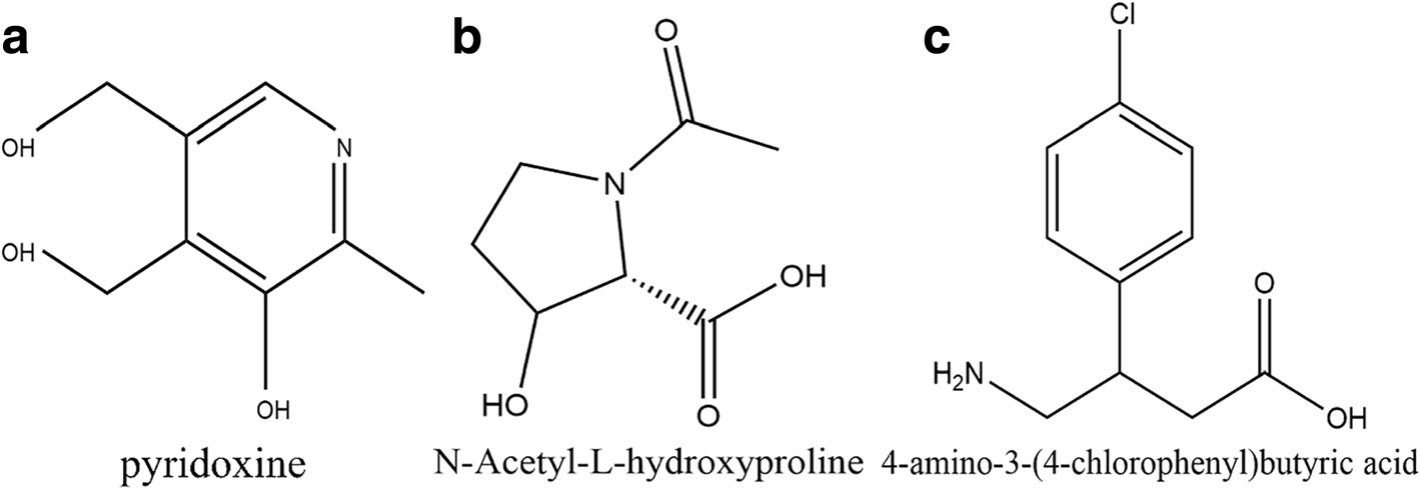
Chemical structures of the drugs. **a** Vitamin B6, **b** N-Acetyl-L-Hydroxyproline and **c** Baclofen analyzed as inhibitors

N-Acetyl-L-hydroxy proline (Hydroxy-L-proline (HYP) analog) [http://www.macklin.cn/search/33996-33-7], 4-amino-3-(4-chlorophenyl) butyric acid (Baclofen) [http://www.macklin.cn/search/1134-47-0] were tested as inhibitors and their inhibitory efficacy compared with Pyridoxine (vitamin B_6_) obtained from Huazhong Pharmaceutical Co., Ltd. (Fig. 1). Varying concentrations of vitamin B_6_, Baclofen and N-acetyl-L-Hydroxyproline were added to both standard growth medium as well as dietary salt growth medium respectively, and these compounds were tested as inhibitors. The compounds were dissolved in water except for Baclofen which was dissolved in Tris-HCl buffer (pH 7.2) prior to being added to growth medium. Both Baclofen and N-acetyl-L-Hydroxyproline were obtained from Shanghai Macklin Biochemical Co. LTD while vitamin B6 from Huazhong Pharmaceutical Co. LTD.

Inhibition efficacy of L-Proline analogs selected was compared with the traditional drug (vitamin B_6_) at varying concentrations and their respective survival proportions obtained. Furthermore, the exacerbating effects of a lithogenic factor (NaOx) were also excogitated.

#### AGT knockdown

A Drosophila nephrolithiasis model was established by using the RNAi line for dAGXT (Tsinghua Fly Center, Beijing, CG3926RNAi, TH02225.N, AT04446p [*Drosophila Melanogaster*]), driven by Actin-GAL4/CyO.

Actin>dAGXT RNAi recombinant line was generated by crossing Actin-GAL4/CyO (☿) x UAS-CG3926 RNAi (*♂*). Actin-GAL4/CyO (☿) flies were crossed into each transgenic CG3926 RNAi (*♂*) flies and upon eclosion, the progenies were then moved out and transferred to the respective diets as their parental lines. Tubules from knockdown candidate flies were dissected and examined for CaOx crystal formation.

#### Validation of RNAi knockdown

Knockdown of target genes relative to parental lines was assessed by quantitative RT-PCR (qRT-PCR). RNA was isolated from five female, and five male adults fly abdomens by using the Trizol® Reagent (Invitrogen). cDNA was generated from approximately 1 μg of RNA by using PrimeScript™ RT reagent Kit with gDNA Eraser (Takara) for each sample and q-PCR performed in One step plus real-time PCR. qRT-PCR primers utilized for the reference gene, β-actin(Act5C) were: 5’-GACTTTGAGCAGGAGATGGC-3′ and 5′- AAGCCTCCATTCCCAAGAAC-3′. While for the target gene, CG3926(dAGXT), the primers used were: 5’-GACGAGTGGAAGGTGGATGT-3′ and 5’-AAACCTTCGGCTTGGTCTTT-3′.

#### Observation of CaOx crystal formation

Adult flies fed on standard growth medium (≥7 days), or dietary growth medium for (24 h – 72 h) were randomly extracted from each group, anesthetized by Carbon dioxide (CO_2_) on standard fly pads. Then Malpighian tubules were dissected out using 0.1 M Hepes buffer, two pairs of forceps and a dissecting microscope (Motic SMZ-161). The Malpighian tubules were then removed and mounted onto a fluorescence microscope (OLYMPUS BX41) to visualize the Malpighian tubules for any crystals formed. After that, the respective images were taken using image pro plus version 6.0.0.260 at a standard magnification of × 60 or processed for further examination of the crystals by Scanning Electron Microscopy (SEM).

SEM and energy dispersive X-ray spectroscopy (EDS) Microanalysis: Qualitative analysis employing EDS is an effective tool in microanalysis. Elemental analysis in SEM was achieved by measuring the energy in conjunction with the intensity distribution of the X-ray signal generated by a focused electron beam. Hexamethyldisilazane (HMDS) [http://www.aladdin-e.com/zh_cn/h106018.html] was used to provide a rapid and low-cost method for the preparation of soft insect tissues for SEM. This procedure was used in place of critical point drying and takes only minutes.

Freshly dissected Malpighian tubules were immersed in physiological saline, then immediately transferred to a solution containing 1% Glutaraldehyde in 0.1 M HEPES, pH seven [http://www.xiyashiji.com/product.php?key=111-30-8]. Allowed to sit in this fixative for 5 min, washed for 5 min in ddH_2_O, then dehydrated using a series of ethanol washes:

- 70% Ethanol – 5 min;
- 85% Ethanol – 5 min;
- 95% Ethanol – 5 min;
- 100% Ethanol – 5 min.

Moreover, finally immersed in HMDS for 5 to 15 min, air dried at room temperature.

These samples were further processed for SEM and EDS studies to analyze the compositions. Microanalyses were performed with a JSM-7100F Field Emission Scanning Electron Microscope, with EDS, operated at an accelerated voltage of 20 kV. Pieces of glass (2 × 2 cm^2^) were cut with a diamond cutter and fixed on carbon support with carbon tapes and to improve conductivity. In order to improve the image contrast, the samples were first coated with gold using JOEL JFC-1600 Auto Fine Coater operating at 18.5 s and carbon tapes evaporated to a thin layer over the sample.

#### Statistical analysis

Data expressed as Mean ± SD values of independent repetitive experiments were analyzed by Analysis of variance (ANOVA) (*p* < 0.05) to estimate the differences between the inhibition efficacy of the drugs tested using the software SAS version 9.4 and GraphPad Prism version 6.01. Lifespan was calculated using log-rank test with the same software.

## Results

We demonstrated the Drosophila model for urolithiasis utilizing knocking-down of the dAGXT gene implicated in the formation of stone effectuating with adult *D. Melanogaster* Malpighian tubules.

### *dAGXT* downregulation

**Fig. 2:**
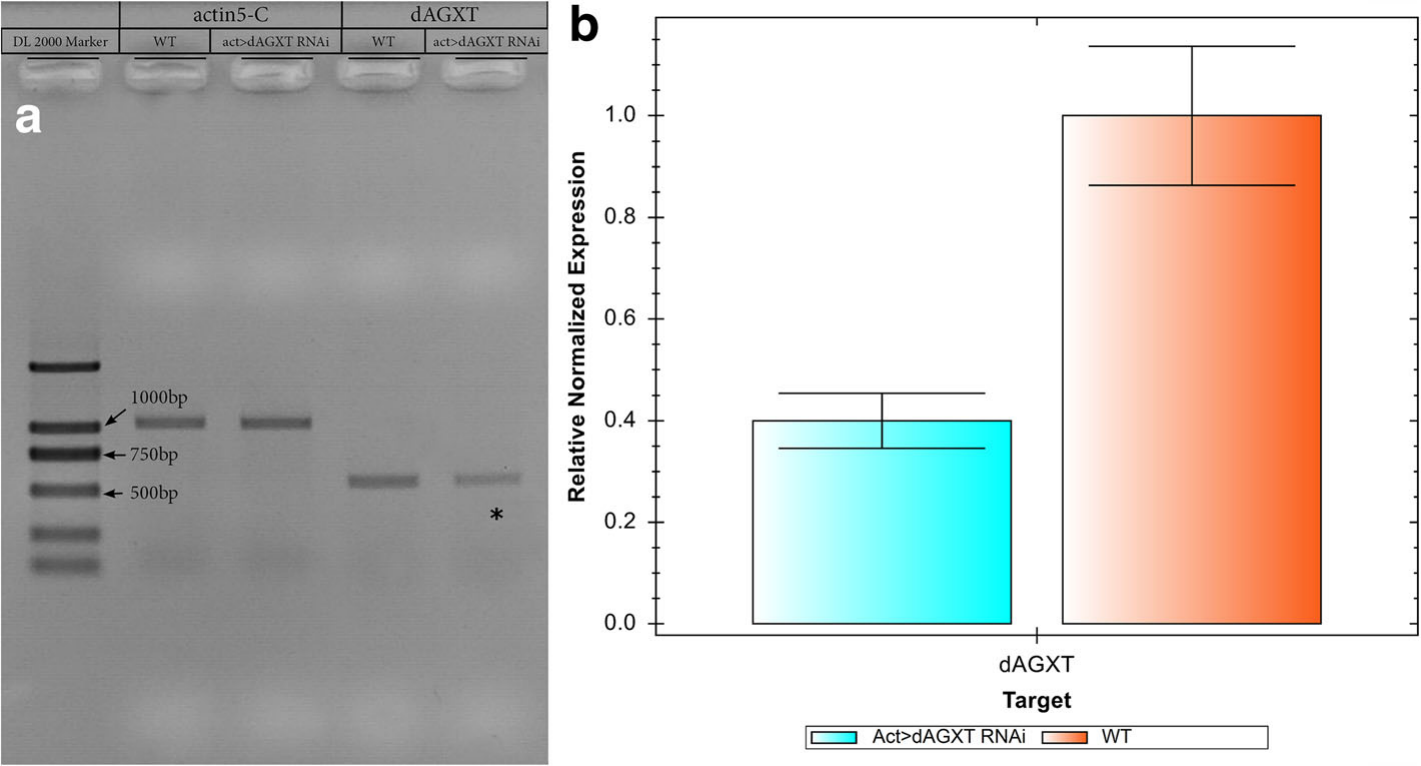
dAGXT (CG3926RNAi) gene downregulation. **a** RT-PCR showing downregulation of the target gene dAGXT (CG3926RNAi). **b** Confirmation of dAGXT downregulation by real -Time qPCR, Relative Normalised Expression = 0.40006 and *P*-Value = 0.0045

To investigate the downregulation of the target gene *dAGXT (CG3926RNAi)*, we carried both RT-PCR and Real-Time qPCR and obtained the relative normalized gene expression calculated according to the ΔΔCt method by utilizing β-actin as the internal control. The normalized gene expression of our target (*dAGXT)* for Actin>dAGXT RNAi was significantly knocked down (*P*-Value = 0.0045) as shown in Fig. 2.

### CaOx crystal formation

**Fig. 3:**
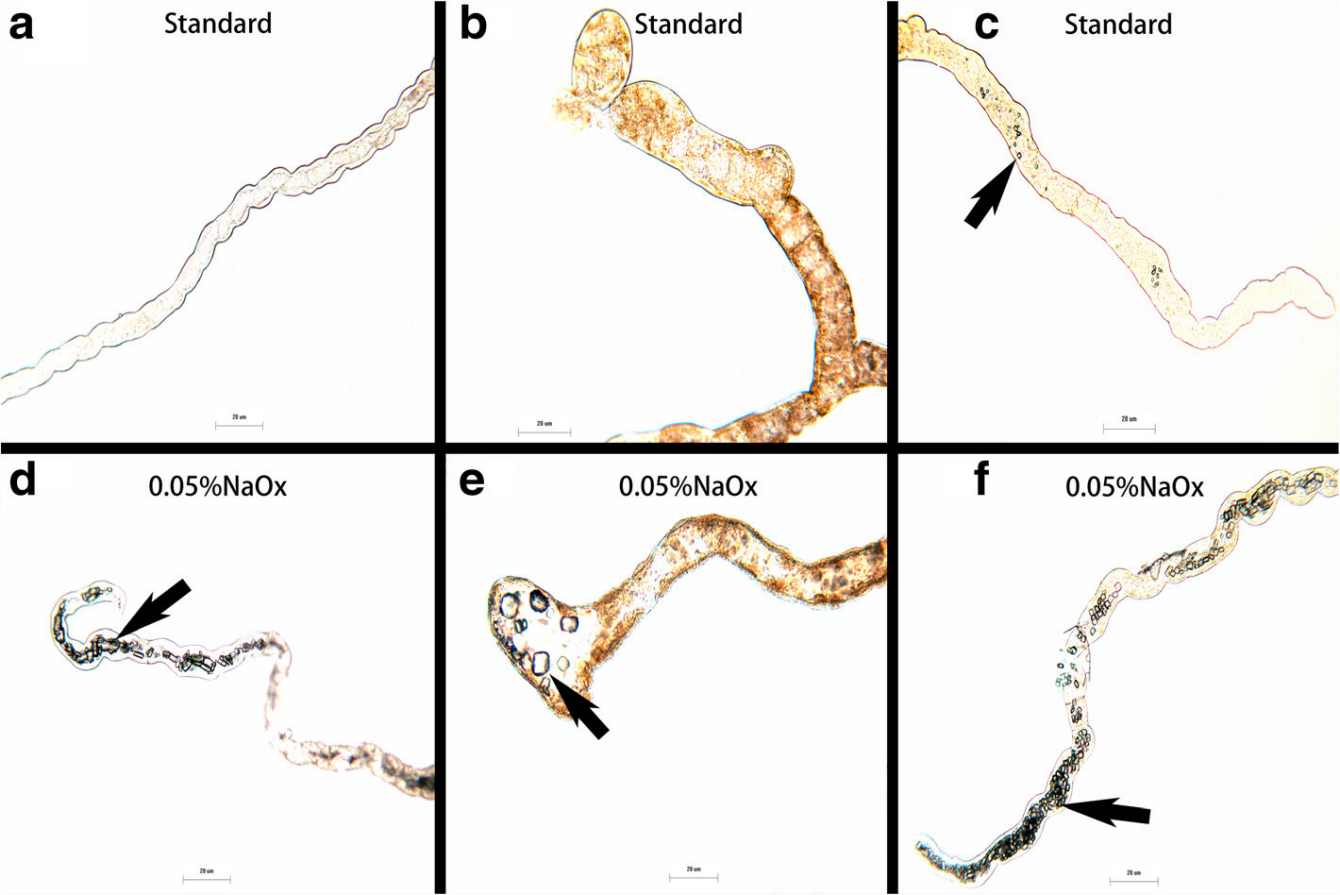
Fluorescence microscope (OLYMPUS BX41) Observation of stone Formation at × 60 magnification. **a** W118 blank control (No stones formed), **b** CG3926RNAi negative control (No stones formed), **c** actin>dAGXT RNAi (stones formed with standard growth medium) **d** W118 (forms stones with (0.05%NaOx) dietary salt growth medium), **e** 3926RNAi (forms stones with 0.05%NaOx dietary salt growth medium), **f** actin>dAGXT RNAi (the amount of stones increased with (0.05%NaOx) dietary salt growth medium as opposed to standard growth medium)

**Fig. 4:**
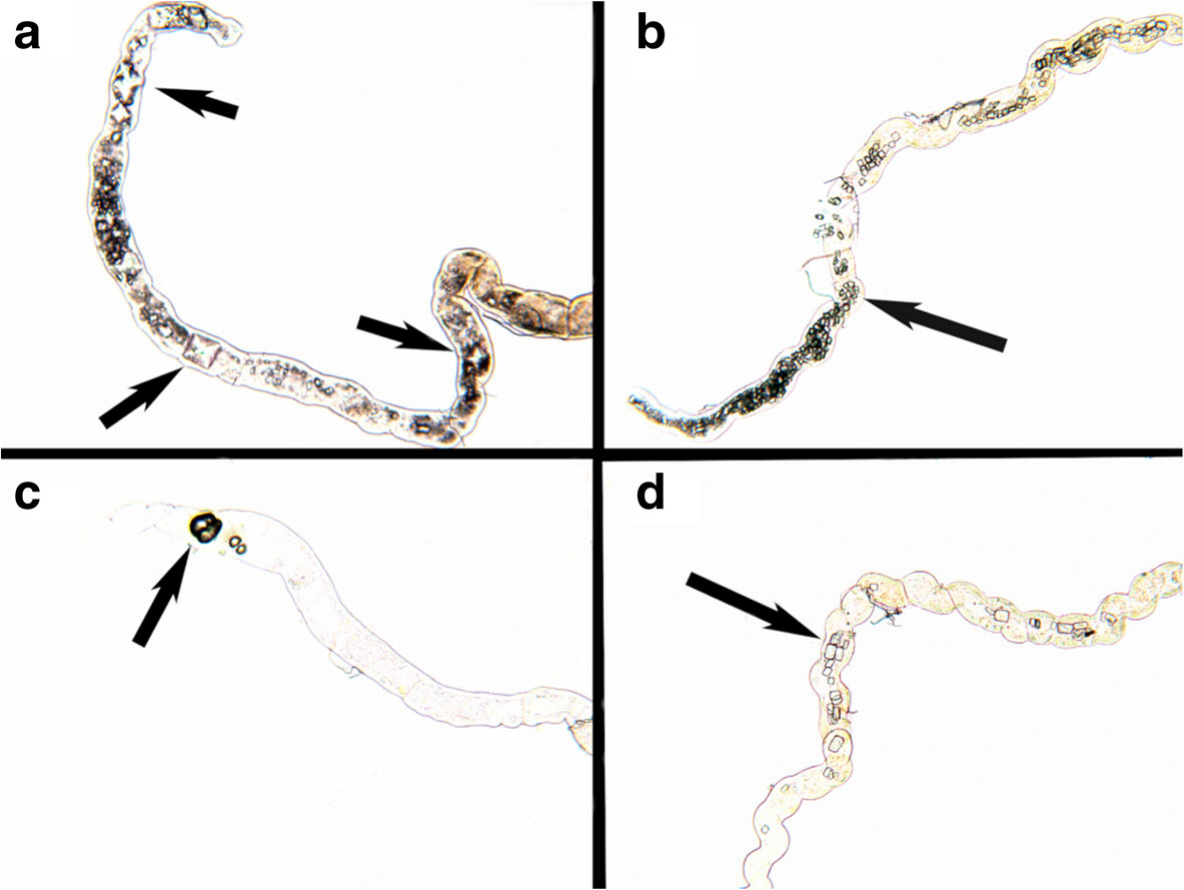
Different forms of CaOx Crystal Formed observed using Fluorescence microscope (OLYMPUS BX41) at × 60 magnification. **a** Dihydrate CaOx (Octahedral), **b** Monohydrate CaOx (long), **c** Monohydrate CaOx (Dumbbell and ovoid). **d** Monohydrate CaOx (Long)

The crystal formation was only observed in Actin>dAGXT RNAi (experimental group) for flies that were only fed on standard growth medium. However, all the three groups that are Control (W^118^), Act-gal4 driven RNAi knockdown (Actin>dAGXT) and the non-driven RNAi line (CG3926RNAi) formed crystals when either of the groups was fed on a diet containing 0.05% NaOx (dietary salt growth medium) as shown in Fig. 3. Both calcium oxalate monohydrate (COM) and calcium oxalate dihydrate (COD) crystals shapes and morphologies formed as shown in Fig. 4.

### Crystals are CaOx

**Fig. 5:**
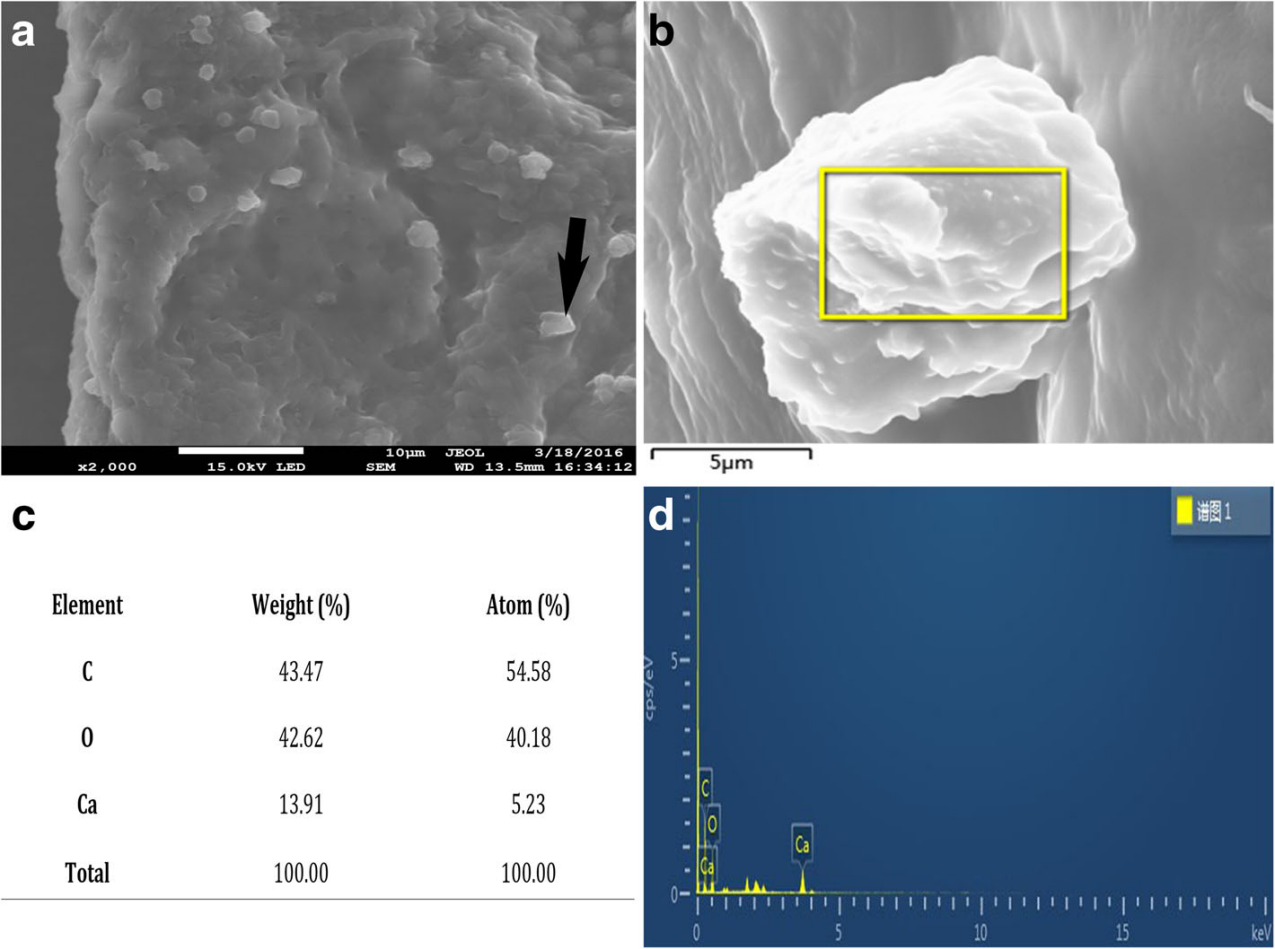
Using SEM and EDS to determine crystal element composition. **a** SEM showing CaOx crystals, **b** EDS CaOx crystal analysis, **c** Table showing EDS analysis for the respective elements of CaOx, **d** EDS analysis graph obtained when the energy emission of CaOx crystal from **b** was being analyzed

To substantiate that the formed crystals were CaOx, SEM and EDS analysis were employed to identify the crystals formed conclusively. Figure 5a shows a segment of the Malpighian tubule with crystals which were then subjected to individual element analysis by examining the energy dispersed by the crystals as shown in Fig. 5b. From the individual weights of the desired elements shown in Fig. 5c and d, the crystals were found to be composed of 13.91% Calcium, 43.47% Carbon and 42.62% Oxygen as shown in Fig. 5.

### Drosophila survival proportions

**Fig. 6:**
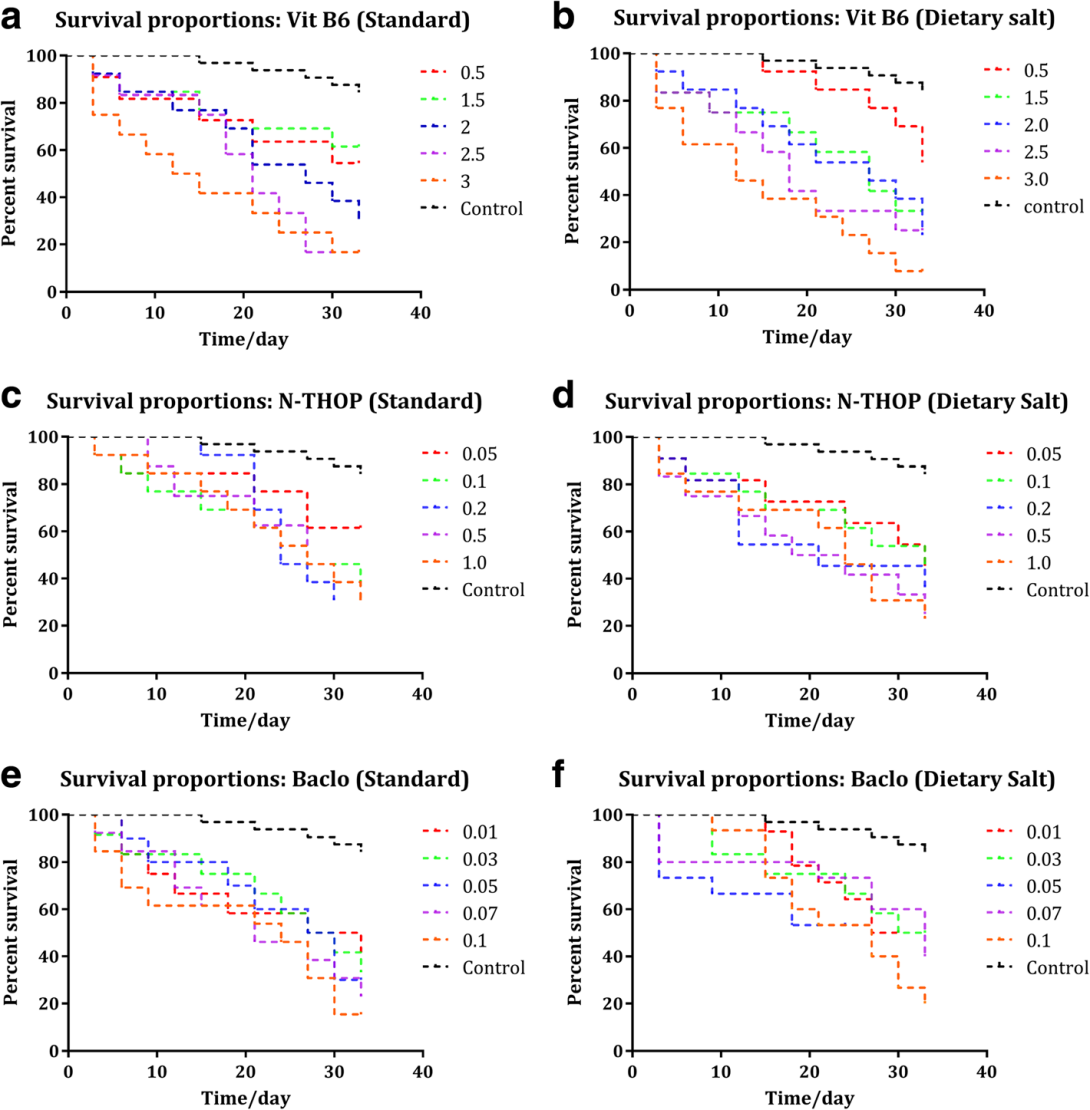
log-rank test survival proportions analyzed at different concentrations. **a** Vitamin B6 survival proportions (Standard), **b** Vitamin B6 survival proportions (Dietary salt), **c** N-Acetyl-L-Hydroxyproline survival proportions (Standard growth medium) **d** N-Acetyl-L-Hydroxyproline (N-THOP) survival proportions (Dietary salt growth medium), **e** Baclofen survival proportions (Standard growth medium) and **f** Baclofen survival proportions (Dietary salt growth medium)

To examine the association between inhibitor dosage and mortality rates, the survival proportions were analyzed at different concentrations of each inhibitor both for standard growth medium and dietary salt growth medium under the same conditions of 25 °C temperature and (40–60) % humidity for twelve-hourly light/dark cycle. The significance of a change in lifespan was measured using the log-rank test which generally reduced with increasing concentrations of the three inhibitors from a normal range of (60–80) days to (35–45) days and for uniformity purposes, all the survival proportions were calculated up to 39 days since some groups no longer had any survivors and the primary objective of carrying out survival tests was to get the best concentration (that’s with good inhibition efficiency and moderate mortality as a result of the inhibitor). Moreover, the best-estimated concentrations of each inhibitor (2% Vitamin B_6_, 0.2% N-Acetyl-L-Hydroxyproline and 0.05% Baclofen) were selected and used for further contrast test among the inhibitors as shown in Fig. 6.

### Inhibition of CaOx crystal formation

**Fig. 7:**
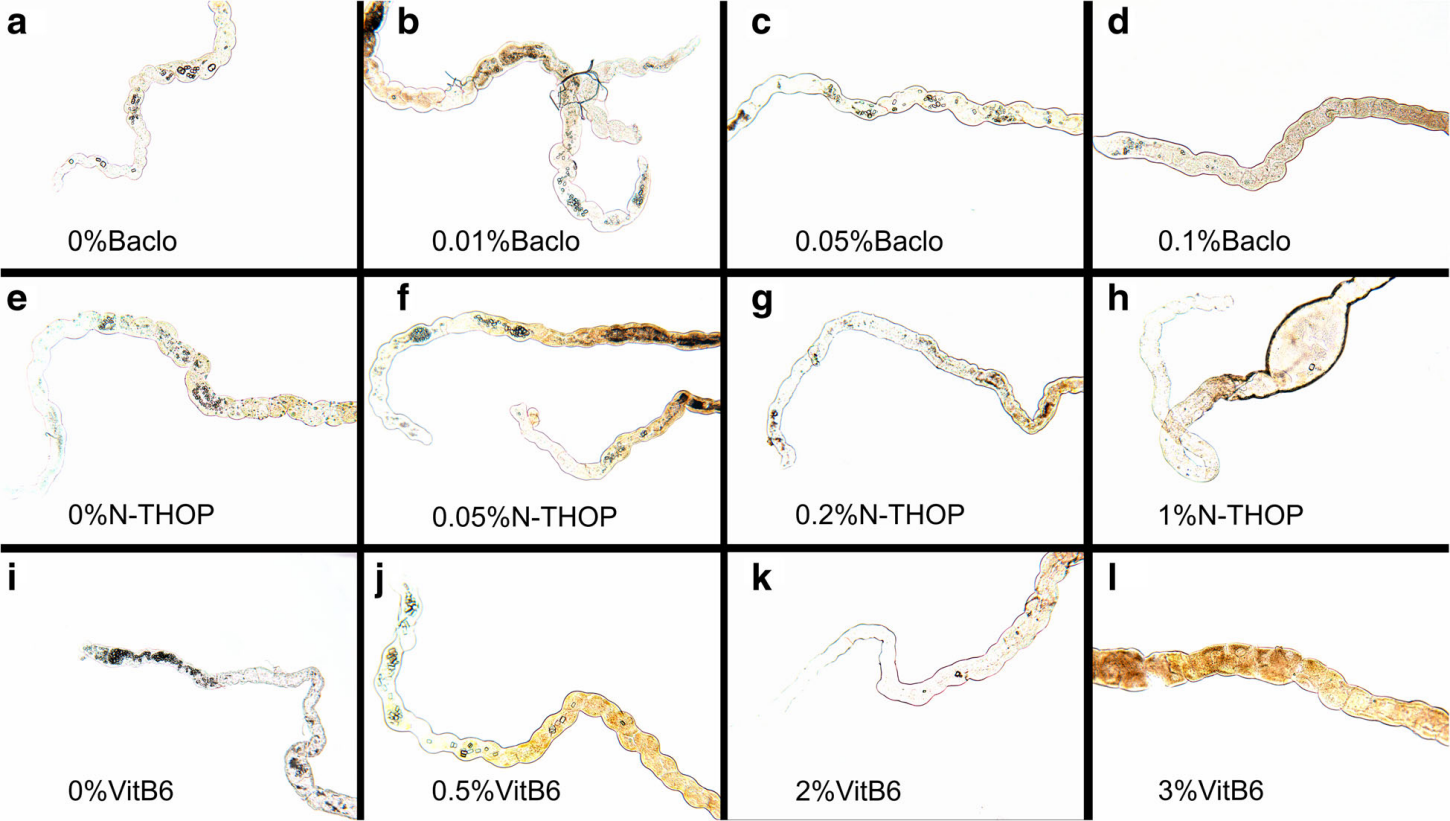
Showing fluorescence microscope images of standard growth medium for the three inhibitors with increasing low, moderate and high concentrations respectively. **a**, **e** and **i** were controls at 0% Baclofen, N-Acetyl-L-Hydroxyproline (N-THOP) Vitamin B_6_ respectively with high numbers of CaOx crystals, **b** Baclofen, 0.01% showed litile inhibition effects and a great deal of CaOx crystals still visible, **c** 0.05% Baclofen, showing slight reduction in the amount of crystals, **d** 0.1% Baclofen with much improved reduction in the amount of CaOx crystals, **f** 0.05% N-Acetyl-L-Hydroxyproline (N-THOP) less or no reduction in the inhibition of CaOx, **g** 0.2% N-Acetyl-L-Hydroxyproline (N-THOP), greatly inhibited the amount of CaOx crystals, **h** 1% N-Acetyl-L-Hydroxyproline (N-THOP) showing significantly reduced amount of CaOx crystals to almost a single crystal, **j** 0.5% Vitamin B_6_ still with a high amount CaOx crystals, **k** 2% Vitamin B6 showing moderate amount CaOx crystals, **l** 3% Vitamin B6 showing hardly any CaOx crystals

**Fig. 8:**
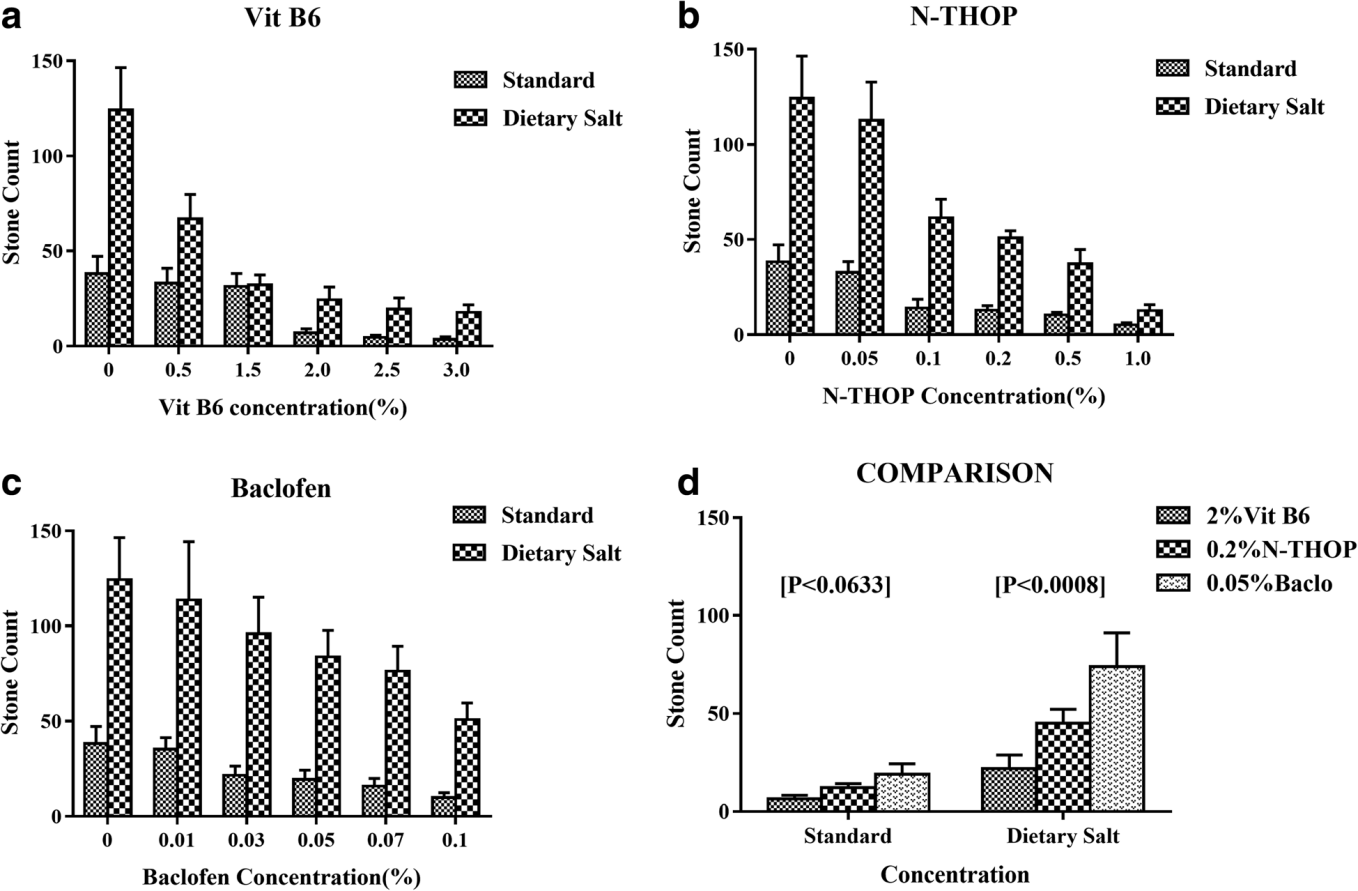
Inhibition efficacy of the three drugs. Data were Mean ± SD of eight independent observations. **a** Vitamin B6 stone count histogram (Traditional drug), **b** N-Acetyl-L-Hydroxyproline (N-THOP) histogram (Experimental inhibitor), **c** Baclofen histogram (Experimental inhibitor). **d** Histogram comparison the best concentration of each inhibitor (2% Vitamin B6, 0.2% N-Acetyl-L-Hydroxyproline (N-THOP) and 0.05% Baclofen)

**Fig. 9:**
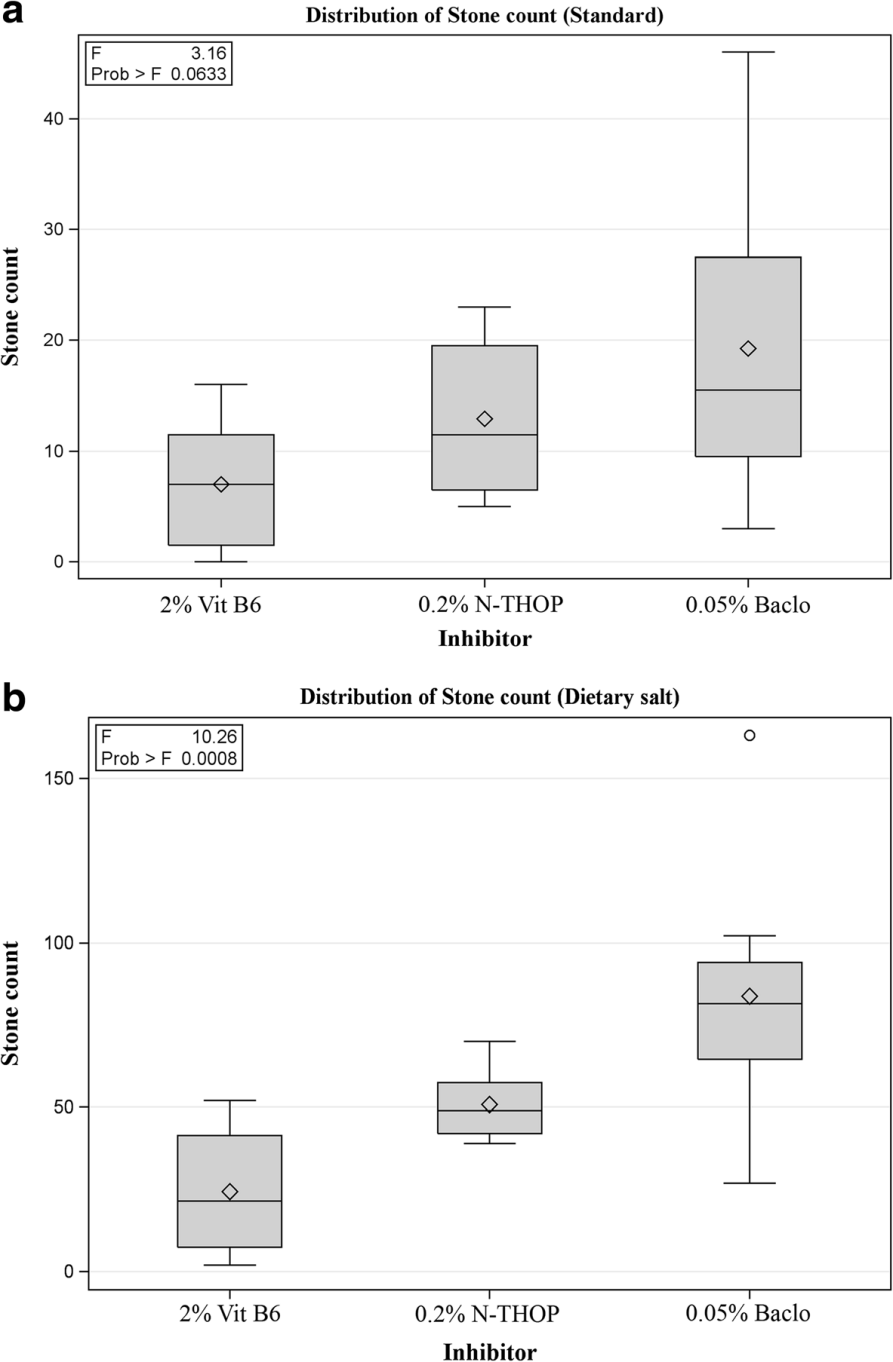
One-way ANOVA boxplot comparing inhibition efficacy. **a** *P*-value for standard growth medium group (Prob > F (the *p*-value) is 0.0633). **b** *P*-value for dietary salt growth medium group substantial statistical significance (Prob > F (the *p*-value) is 0.0008)

**Fig. 10:**
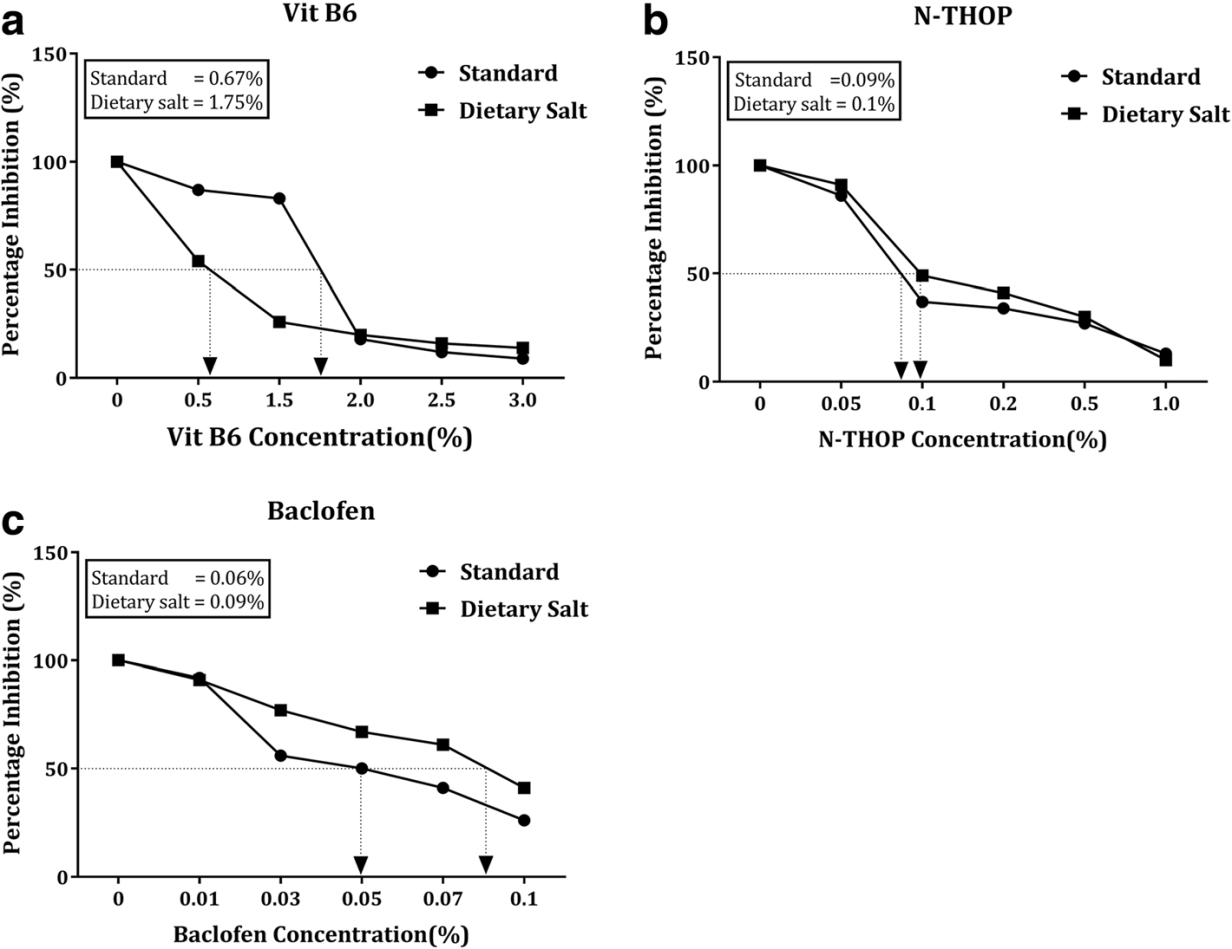
Percentage inhibition curves of stone formation. **a** Vitamin B6 percentage inhibition curves, generally higher concentration of vitamin B6 were required to obtain a substantial inhibition especially for standard growth medium group as opposed to the two Hydroxy-L-proline (HYP) analogs, **b** N-Acetyl-L-Hydroxyproline (N-THOP) percentage inhibition curves, produced consistent inhibition curves with both standard and dietary salt growth medium growth media respectively. **c** Baclofen percentage inhibition curves resulted in CaOx crystal inhibition with the least concentration of inhibitor added to the growth medium

To compare the efficacy of the inhibitors, average stone counts at different concentrations across the three groups were analyzed to get the general picture and later the best concentration was ascertained. There was a significant decrease in the number of stones with an increase in the concentration of each inhibitor as shown in Figs. 7 and 8. Vitamin B_6_ still proved to be the best inhibitor though at very high concentrations that are ≥2% Vitamin B_6_ as shown in Figs. 8 and 9. However, at a lower concentration, Vitamin B_6_ had low inhibition especially for the dietary salt growth medium as opposed to N-Acetyl-L-Hydroxyproline which was observed to have consistent inhibition function for both growth medium as shown in Fig. 8. This is supported by the IC_50_ estimates where Vitamin B_6_ required approximately 0.6 and 1.8% (that is standard and dietary salt growth medium respectively) to produce the same inhibition as 0.1% N-Acetyl-L-Hydroxyproline or 0.06 and 0.1% Baclofen (standard and dietary salt growth medium respectively) as shown in Fig. 10.

By ANOVA, we noted that the value of P was not significant for the standard growth medium group that is Vitamin B_6_ against N-Acetyl-L-Hydroxyproline, and VitB_6_ against Baclofen (*P* < 0.0633). But despite this, it is within a confidence interval of 90% as described by the distribution of stone count where the control group (Vitamin B_6_) was showing the Mean ± SD values of (7.00 ± 6.05) compared N-Acetyl-L-Hydroxyproline (12.88 ± 7.00) and Baclofen (19.25 ± 14.14) respectively as shown in Figs. 6 and 7. On the contrary, there was a substantial statistical significance in the stone count distribution for the dietary salt growth medium group (*p* < 0.0008). However, vitamin B_6_ required higher concentrations to produce the same result of CaOx crystal inhibition as our L-Proline analogs at very low concentrations. Hence, owing to their low toxicity level coupled with very their high inhibition efficacy at very low concentration, N-Acetyl-L-Hydroxyproline and Baclofen could provide potent antilithiatic alternatives.

## Discussion

Both technologies and techniques for the surgical treatment of PH have tremendously advanced in the last two decades. There is progress with the ongoing therapeutic revolution for the management of PH with RNA modulation through Dicer-substrate small interfering RNAs (DsiRNAs) targeting hydroxyacid oxidase 1 (HAO1) mRNA that encodes glycolate oxidase (GO), to reduce the hepatic conversion of glycolate to glyoxylate. Results obtained in the preclinical mouse model of PH1were promising and clinical trials are going on [29]. Although the concept of Dicer modulation is promising to be efficient and safe in decreasing hepatic GO which in turn can lead to normalization of urine oxalate levels and reduces CaOx deposition, interest of crystallization inhibitors such Hydroxy-L-Proline analogs is still very necessary to realize breakthroughs that can aid in the development of efficient and effective medications for either prevention or treatment. Conventional medical therapy for nephrolithiasis only alters gross urinary constituents to lessen the risk of stone formation yet stone formation is actually the endpoint of a complex pathophysiologic process which is still poorly understood, therefore, interventions such Hydroxy-L-Proline analogs that directly target these fundamental mechanisms with very low side effects or coupled with either limited short-term or long-term efficacy provide unique opportunity to develop novel therapies for nephrolithiasis [26]. Other therapies currently in use include Pyridoxine supplementation in PH type 1 patients and, later liver and/or kidney transplantation [30, 31]. Since most of the current treatment options have limited success [32], there is a great need for new cost-effective, efficacious and readily deployable therapeutic master plans.

We, therefore, considered small nontoxic molecules (Hydroxy-L-Proline analogs) that will inhibit PRODH2 and its activity. The inhibition of this novel drug target has the potential to alleviate the high levels of glyoxylate and oxalate in all three forms of PH in patients [13]. Previous studies mainly focused on in vitro studies, and some chemicals have been identified as being effective in inhibiting PRODH2 [13]. However, the application of *D. Melanogaster* model was able to analyze the inhibitory efficacy of Hydroxy-L-Proline analogs in vivo studies hence lessening limitations of this novel drug target for future in vivo research.

Fortunately, the *D. Melanogaster* has now emerged as a compelling translational model of human nephrolithiasis with a diversity of functional and pragmatic advantages. The Malpighian tubules of this versatile invertebrate correspond to the remainder of the human nephron along with collecting duct. The preservation of the genetic composition together with transporter protein structure, including the similarities of physiologic function of the Malpighian tubules, has aided the development of several Drosophila stone models [3, 4, 26]. Most recently, *D. Melanogaster* models of calcium oxalate nephrolithiasis have been described providing a criterion to study and understand mineralization in invertebrates [27].

**Fig. 11:**
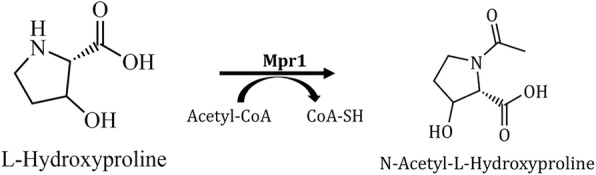
Detoxification of L-Hydroxyproline by converting into N-acetyl L-Hydroxyproline through acetylation using Acetyltransferase Mpr1

So, we used *D. Melanogaster* as the model to develop novel therapies for nephrolithiasis. When Actin>dAGXT RNAi recombinant lines’ progenies were collected and examined, they confirmed that the dAGXT gene was successfully downregulated. From these results, we have successfully knocked down the dAGXT gene in *Drosophila*, and the stone crystals could be visualized in the Malpighian tube when *Drosophila* fed on standard growth medium (see Fig. 3). The analysis of stone composition shows that almost all of them are calcium oxalate stones. Our study conforms with Summitt and colleagues’ findings which stated that blocking the activity of PRODH2 is a potential avenue for the treatment of PH, and henceforth inhibitors of PRODH2 activity would be of keen interest to researchers [13]. Previous in vitro studies showed that Tetrahydro-2-furoic acid (THFA) was more effective than the other analogs in preventing PRODH2 activity because the oxygen atom on the ring of THFA replaces the nitrogen atom in proline, and thus prevents the ring from opening during catalysis [13]. However, THFA is not suitable for application because it is toxic to people’s health. Furthermore, a diversity of synthetic proline analogs dependent on ring substitutions with alkyl and aromatic groups, incorporation of heteroatoms into the ring, and expansion or contraction of the proline have been developed ring [33]. These are derivatives, antagonists, and mimetics of proline that are usually promising candidates for tuning the biological, pharmaceutical or physicochemical properties of naturally occurring or de novo designed peptides [33]. However, most of these analogs are toxic to cells. Nevertheless, some of these L-Proline analogs such as L-Hydroxyproline, THFA, Azetidine-2-carboxylic acid (AZC) could be detoxified when a nitrogen atom replaces the oxygen atom on the furan ring. For example, N-acetyltransferase Mpr1 could detoxify L-Hydroxyproline by converting into N-acetyl -L-Hydroxyproline (see Fig. 11) [23, 24, 33].

Meanwhile, nitrogen atom on the furan ring of N-acetyl-L-Hydroxyproline replace oxygen atom that will significantly reduce its toxicity, and this can be affirmed in *Drosophila Melanogaster* (see Fig. 6).

Therefore, we have chosen analogs of this particular structure. In this paper, we chose N-acetyl-L-Hydroxyproline and Baclofen derived from hydroxyprolines (HOPs) [33] to study their abilities to inhibit the stone formation and their possible toxicity in *Drosophila Melanogaster*. From the results, we found these inhibitors showing a significant inhibitory effect on the stone formation at low concentration when compared with the effect of vitamin B_6_ as shown in Figs. 8d, 9 and 10.

In relation to toxicity levels, L-Proline analogs had lower toxicity levels compared to Vitamin B_6_ during the survival proportion analysis as shown in Fig. 6. This may be due to the high concentration levels required to obtain the same inhibition efficacy as the low doses of L-Proline analogs. Secondly, Pyridoxine (VitB_6_) has also been associated with some cases of peripheral neuropathy, dermatoses, photosensitivity, dizziness, and nausea have been reported with long-term mega doses of pyridoxine over 250 mg/day. A few cases of neuropathy appear to have been caused by chronic intake of 100 to 200 mg/day [34–36]. Therefore, the higher dose of Vitamin B_6_ (0.6 and 1.8% standard and dietary salt growth medium respectively) was required to produce the same inhibition as 0.1% N-Acetyl-L-Hydroxyproline or 0.06 and 0.1% (standard and dietary salt growth medium respectively) Baclofen might be strongly related to the toxicity levels as shown in Fig. 6.

In a nutshell, the analogs acting as inhibitors of proline dehydrogenase have already been identified, laying the foundation for developing novel therapeutics to block the hydroxyproline degradation pathway and reducing the glyoxylate burden in PH patients [13].

## Conclusion

The results of this research show that addition of Hydroxy-L-Proline analogs to growth medium resulted in the reduction of calcium oxalate (CaOx) crystals formation. Therefore these analogs show promise as potential inhibitors for oxalate reduction in Primary Hyperoxaluria therapy. However, these are only the results of *Drosophila Melanogaster* model, to further clarify their efficacy and toxicity, similar studies in mammals such as mouse models need to be carried out to expound the effects of these two drugs on in vivo metabolism.

## Abbreviation

AGT: Alanine-glyoxylate aminotransferase
CaOx: Calcium oxalate
EDS: Energy dispersive spectroscopy
ESRD: End-stage renal disease
GRHPR: Glyoxylate reductase hydroxy pyruvate reductase
HOGA1: 4-hydroxy-2-oxoglutarate aldolase
N-THOP: N-Acetyl-L-Hydroxyproline
PH: Primary hyperoxaluria
PRODH2: Proline dehydrogenase 2
SEM: Scanning Electron Microscopy
THFA: Tetrahydrofuroic acid
